# The NSUN5 gene rs1880948 A>G polymorphism and neuroblastoma risk in Chinese children

**DOI:** 10.3389/fonc.2025.1655312

**Published:** 2025-12-19

**Authors:** Zijie Ye, Huijuan Zeng, Manna Zheng, Chao Hu, Jing Pan, Jiliang Yang, Tianbao Tan, Chunlei Zhou, Jing He, Yan Zou, Tianyou Yang

**Affiliations:** Department of Pediatric Surgery, Guangzhou Women and Children’s Medical Center, Guangzhou Medical University, Guangdong Provincial Clinical Research Center for Child Health, Guangzhou, China

**Keywords:** cancer, neuroblastoma, NSUN5, SNP, 5-methylcytosine modification

## Abstract

**Background:**

Neuroblastoma (NB) is the most common extracranial solid tumor in children. The 5-methylcytosine (m5C) modification gene NSUN5 polymorphisms may serve as promising molecular markers for identifying populations susceptible to NB.

**Method:**

TaqMan probes were used to genotype NSUN5 single nucleotide polymorphisms (SNPs) in 402 NB patients and 473 healthy controls. A logistic regression model was applied to calculate the odds ratio (OR) and 95% confidence interval (CI) to evaluate the association between genotype polymorphisms and NB susceptibility. The analysis was further stratified by age, gender, tumor origin site, and clinical stage.

**Conclusion:**

In summary, our study indicates that the selected NSUN5 rs1880948 A>G polymorphisms may not be associated with neuroblastoma susceptibility. However, further studies with larger sample sizes and additional potentially functional polymorphisms are needed to validate these results.

## Introduction

Neuroblastoma (NB) is an extracranial solid malignant tumor that originates from precursor cells of the sympathetic nervous system, and it predominantly affects children under 5 years of age (1, 2). Although accounting for only 7-8% of childhood malignancies, the incidence of NB in children of China is about 7.7 per million, accounting for about 15% of childhood malignancies deaths (3). Although low-risk patients have higher therapeutic effect, the 5-year survival rate of high-risk NB patients is less than 50% (4, 5). Although some patients with mild or no treatment show spontaneous regression, more than half of high-risk neuroblastoma patients die even with multimodal therapy such as surgery, chemotherapy, radiotherapy, and immunotherapy (6). Due to the limited means of treatment, the etiology and pathogenesis of NB are still unclear, which poses a serious challenge to the treatment of NB. Therefore, it is critical to uncover valuable genetic changes in neuroblastoma to screen high-risk individuals and explore potentially effective treatments.

Various genetic alterations have been identified in neuroblastoma, including amplification of MYCN, mutations in ALK, and segmental chromosomal changes (7). Amplification of the MYCN gene has been found in most malignant neuroblastomas, occurring in about 20% of the all cases. Segmental chromosomal loss of the distal short arm of chromosome 1 (1p) and the long arm of chromosome 11 (11q) is associated with poor survival in patients (8). Germline gain-of-function mutations in ALK is the main drivers of most familial neuroblastoma (9, 10). Additionally, other relevant gene mutations have been found in neuroblastoma, such as TERT, ATRX, TP53, and RAS (11–14). However, these cannot fully represent all genetic risk factors for neuroblastoma. More research is needed to discover other genetic mechanisms.

Single nucleotide polymorphisms (SNPs) are the most common type of genetic variation among individuals (15). In a vast number of genome-wide association studies (GWASs), it has been investigated that many SNPs are associated with the incidence of neuroblastoma or the success of therapy, such as genetic variants in LIN28B, AXIN2, LMO1, BARD1, CDK1NB, CASC15, SMARCA4, CHEK2, BRCA1, and TP53 (16–19). These studies demonstrate that genetic variants play a significant role in the occurrence and progression of NB and lay the foundation for potential mechanisms and treatment strategies.

Currently, epigenetics is a focal point in scientific research, particularly in understanding its role in regulating cancerous characteristics through the modification of RNA. 5-methylcytosine (m5C) is a highly concentrated epigenetic modification, and as more researchers learn about RNA m5C modification, they are finding many targets in multiple RNA species and in a variety of organisms (20, 21). Modification of RNA m5C has been implicated in many human cancers, including leukemia, lung cancer, gastric cancer, liver cancer, and gynecological tumor (22–24). Demethylases can remove methyl groups from cytosine, restoring its original state (25). The binding proteins recognize and bind to m5C, influencing gene expression and RNA function (26).

Enzymes regulating m5C levels of RNAs can be functionally categorized as methyltransferases (writers), demethylases (erasers) and binding proteins (readers) (27). Methyltransferases add a methyl group to cytosine to form m5C, including the NOL1/NOP2/Sun RNA methyltransferases (NSUN) (28). The NSUN family functions as the primary enzymes mediating m5C RNA methylation, employing S-adenosylmethionine as a methyl donor to transfer methyl groups and thereby generate m5C (29). Multiple studies have been performed that NSUN5 is overexpressed in various types of cancers including gastric cancer, hepatocellular cancer, renal cancer, colorectal cancer (30–33). Together, these findings suggest that the NSUN5 gene may serve as a potential therapeutic target and prognostic marker. Nevertheless, the relationship between NSUN5 polymorphisms and neuroblastoma susceptibility remains unclear, warranting further investigation to elucidate their role in neuroblastoma. Putative functional potentials SNPs located in the 5’-flanking region, exon, 5’- untranslated region (5’ UTR), which might affect transcription activity or binding capacity of the microRNA binding site (34). The NSUN5 rs1880948 A>G polymorphism is located in the region near the 5’ UTR, potentially affecting the functional output of the gene. However, few studies have focused on neuroblastoma. Here, we performed a hospital-based case-control study using data from 402 neuroblastoma patients and 473 control subjects to evaluate the association between the NSUN5 gene (rs1880948 A>G) polymorphism and neuroblastoma risk in Chinese children.

## Materials and method

### Study subjects

This work was conducted with the approval of the Institutional Review Board of Jiangsu Province, with 402 neuroblastoma patients registered in Children’s Hospital of Nanjing Medical University and 473 age- and gender-matched healthy controls involved in previous studies from January 30, 2008 to November 2, 2021. The ethnicity of all participants was recorded in their medical records at the time of hospital admission, and all subjects were classified as Han Chinese. The study was approved by the Institutional Review Committee of Nanjing Medical University Children’s Hospital (approval number: 202112141-1), and all procedures were conducted in accordance with ethical standards and regulatory requirements. All participants signed informed consent.

### Polymorphism selection and genotyping

Only one SNP in the NSUN5 gene (rs1880948 A>G) was chosen and genotyped in this study. Potentially functional NSUN5 SNPs were obtained from the NCBI dbSNP and SNPinfo Web databases. The polymorphism resides within the intronic region of the NSUN5 gene and is predicted to function as a potential transcription factor binding site. SNPs with low linkage disequilibrium (LD) relative to other SNPs were chosen based on an R² cutoff of <0.8. Genomic DNA was isolated from the samples, and SNPs were genotyped using TaqMan probe real-time PCR. The TaqMan probes, which are specifically designed and validated for the target SNPs, contain a fluorescent reporter and a quencher dye. We analyzed repeated genotyping by randomly selecting 10% of samples from both cases and controls. A 100% consistency was achieved.

### Statistical analysis

The study performed χ2 goodness-of-fit test to detect the deviations from Hardy-Weinberg equilibrium (HWE) from Hardy-Weinberg equilibrium in genotype frequencies of the polymorphism in the control group. Two-sided χ2 test was used to determine differences in the frequency distribution of age, sex, and genotypes between the case and control groups. We conducted multivariate logistic regression models to estimate the association between the NSUN5 gene rs1880948 A>G polymorphism and neuroblastoma susceptibility by computing odds ratios (OR) and 95% confidence intervals (CI). Adjusted ORs were calculated using multivariate analysis adjusting for age and gender. All statistical analyses were performed using SAS software (version 10.1; SAS Institute, Cary, NC). Statistical significance was considered at P < 0.05.

## Results

### Population characteristic

Our research population consisted of 402 neuroblastoma patients and 473 cancer-free controls. The demographic characteristics of all patients are shown in **Table 1**. There were no significant differences in age (P = 0.100) and sex (P = 0.987) between the two groups. As for the site of tumor origin, 93 cases (23.13%), 167 cases (41.54%), 120 cases (29.85%) and 18 cases (4.28%) of neuroblastoma occurred in the adrenal gland, retroperitoneal region, mediastinum, and other areas, respectively. However, the origin of 4 neuroblastoma tumors (1.00%) could not be determined due to insufficient samples. In addition, based on the INSS standard (35), 108 (26.87%), 63 (15.67%), 59 (14.68%), 104 (25.87%), and 2 (0.50%) cases are classified into stage I, II, III, V, and 4s, while 66 cases (16.42%) are classified as NA (not available) due to lack of information.

**Table 1:** Demographic characteristics of neuroblastoma patients and cancer-free controls from Jiangsu province.

Variables	Cases (N = 402)		Controls (N = 473)		*P* ^a^
	No.	%	No.	%	
Age range, month	0.033-168.00		0.367-168.00		
Mean ± SD	40.99 ± 35.49		40.88 ± 29.76		0.962^b^
Age					0.100
≤18 months	139	34.58	139	29.39	
>18 months	263	65.42	334	70.61	
Gender					0.987
Female	191	47.51	225	47.57	
Male	211	52.49	248	52.43	
Sites of origin					
Adrenal gland	93	23.13	/	/	
Retroperitoneal region	167	41.54	/	/	
Mediastinum	120	29.85	/	/	
Other region	18	4.48	/	/	
NA	4	1.00	/	/	
INSS stages					
I	108	26.87	/	/	
II	63	15.67	/	/	
III	59	14.68	/	/	
IV	104	25.87	/	/	
4s	2	0.50	/	/	
NA	66	16.42	/	/	

SD, standard deviation; NA, not available.

a Two-sided χ^2^ test between neuroblastoma patients and cancer-free controls.

b t test between neuroblastoma patients and cancer-free controls.

### NSUN5 gene rs1880948 A>G polymorphism with neuroblastoma risk

**Table 2** presents the frequency distribution of genotypes of NSUN5 gene SNP between the case and control groups, as well as the association with neuroblastoma risk. Power analysis indicates that the study has sufficient statistical power to detect a significant association, if one exists. The frequency distribution of genotype of our observations agrees with the Hardy-Weinberg equilibrium (HWE > 0.05) in the control group, supporting the validity of the genetic data and ensuring the robustness of subsequent association analyses. The genotype frequency distribution of the NSUN5 gene rs1880948 A>G polymorphism was as follows: 42.29% (AA), 45.27% (AG) and 12.44% (GG) in the patients, and 38.48% (AA), 46.51% (AG) and 15.01% (GG) in the controls. Statistical analysis found no correlation between the genotype of NSUN5 and neuroblastoma susceptibility, even when age and gender were adjusted for.

**Table 2:** *NSUN5* rs1880948 A>G polymorphism and neuroblastoma risk in children from Jiangsu province.

Genotype	Cases (N = 402)	Controls (N = 473)	P^a^	Crude OR (95% CI)	P	Adjusted OR (95% CI)^b^	P^b^
rs1880948 (HWE = 0.735)							
AA	170 (42.29)	182 (38.48)		1.00		1.00	
AG	182 (45.27)	220 (46.51)		0.89 (0.67-1.18)	0.407	0.89 (0.67-1.18)	0.407
GG	50 (12.44)	71 (15.01)		0.75 (0.50-1.15)	0.185	0.75 (0.50-1.15)	0.185
Additive			0.171	0.87 (0.72-1.06)	0.171	0.87 (0.72-1.06)	0.170
Dominant	232 (57.71)	291 (61.52)	0.252	0.85 (0.65-1.12)	0.252	0.85 (0.65-1.12)	0.252
AA/AG	352 (87.56)	402 (84.99)		1.00		1.00	
GG	50 (12.44)	71 (15.01)	0.272	0.80 (0.55-1.19)	0.273	0.80 (0.54-1.19)	0.272

OR, odds ratio; CI, confidence interval; HWE, Hardy-Weinberg equilibrium.

a χ^2^ test for genotype distributions between neuroblastoma cases and cancer-free controls.

b Adjusted for age and gender.

### Stratification analysis

We further explored the association between the NSUN5 gene rs1880948 A>G polymorphism and neuroblastoma susceptibility based on age, gender, origin sites, and clinical stages. As shown in **Table 3**, when compared with the AA genotype, the AG/GG genotypes of the NSUN5 rs1880948 A>G polymorphism have no correlation with the age, gender, site of tumor, or clinical stages.

**Table 3:** Stratification analysis for the association between *NSUN5* rs1880948 A>G polymorphism and neuroblastoma susceptibility.

Variables	rs1880948 (cases/controls)		OR (95% CI)	*P*	AOR (95% CI)^a^	*P* ^a^
	AA	AG/GG				
Age, month						
≤18	55/54	84/85	0.97 (0.60-1.57)	0.902	0.97 (0.60-1.57)	0.897
>18	115/128	148/206	0.80 (0.58-1.11)	0.183	0.80 (0.58-1.11)	0.183
Gender						
Females	85/88	106/137	0.80 (0.54-1.19)	0.267	0.80 (0.54-1.19)	0.267
Males	85/94	126/154	0.91 (0.62-1.32)	0.602	0.91 (0.62-1.32)	0.607
Sites of origin						
Adrenal gland	38/182	55/291	0.91 (0.58-1.42)	0.667	0.90 (0.58-1.42)	0.664
Retroperitoneal	74/182	93/291	0.79 (0.55-1.12)	0.186	0.79 (0.55-1.12)	0.185
Mediastinum	49/182	71/291	0.91 (0.60-1.36)	0.637	0.91 (0.60-1.36)	0.635
Others	7/182	11/291	0.98 (0.37-2.58)	0.972	0.98 (0.37-2.57)	0.964
Clinical stages						
I+II+4s	69/182	104/291	0.94 (0.66-1.35)	0.745	0.97 (0.67-1.38)	0.838
III+IV	72/182	91/291	0.79 (0.55-1.13)	0.201	0.79 (0.55-1.14)	0.211

OR, odds ratio; CI, confidence interval; AOR, adjusted odds ratio.

a Adjusted for age and gender, omitting the corresponding stratify factor.

## Discussion

Neuroblastoma is a common malignant tumor, and high-aggressiveness neuroblastoma often progresses rapidly, leading to poor prognosis and high recurrence rates. The pathogenesis of neuroblastoma remains unclear due to the involvement of multiple factors. An increasing number of studies suggests that cancer development is an interactive phenotype driven by a combination of genetic mutations and epigenetic mechanisms (36). In recent years, it has been reported that epigenetic changes, including DNA methylation, RNA methylation, histone modification, and non-coding RNAs, can promote the progression of cancer. RNA modifications play a crucial role in cancer, especially m5C and m6A modifications. Furthermore, accumulating evidence implicates the role of RNA m5C in tumorigenesis, including gastric cancer (37), prostate cancer (38), hepatocellular cancer (24), lung cancer (39), bladder cancer (40), and leukemia (41). In humans, m5C RNA modification is catalyzed by the NOL1/NOP2/sun (NSUN) family and DNA methyltransferase 2 (DNMT2) (27). Abnormal transcription of the NSUN5 gene can regulate cell ferroptosis, thereby promoting the occurrence and development of tumors (42, 43). The NSUN5 gene is a commonly mutated gene in many human cancers. In glioblastoma, NSUN5 loss generates an unmethylated status at the C3782 position of 28S rRNA, resulting in an anti-tumor effect. NSUN5 is markedly upregulated in head and neck squamous cell carcinoma and acts as a promoter of colorectal cancer by inducing cell cycle arrest (28). NSUN5 facilitates HCC development by targeting the ZBED3/Wnt/β-catenin signaling pathway (44). NSUN5 found an increase in the number of copies in some cancers.

From the perspective of genetic alterations, NSUN5 may influence tumorigenesis through both somatic mutations and germline polymorphisms. In somatic contexts, rare mutations in NSUN5 have been reported in various cancers, including hepatocellular carcinoma and glioblastoma, which can alter RNA methylation patterns and downstream signaling pathways, promoting tumor progression or affecting tumor suppressor functions (31, 45). These somatic changes are often of high functional impact, but their prevalence in neuroblastoma remains largely unexplored. At the germline level, the NSUN5 rs1880948 polymorphism may act through regulatory mechanisms rather than directly altering protein structure. This variant could affect transcription factor or microRNA binding and thereby influence NSUN5 expression, a mechanism also reported for other gene SNPs (46).However, the important role of NSUN5 gene polymorphisms in neuroblastoma is still unclear. Currently, there is limited research on the association between RNA modification gene polymorphisms and susceptibility to neuroblastoma. We, therefore, focused on whether m5C modification gene NSUN5 polymorphisms have an impact on genetic susceptibility to neuroblastoma. More studies are still needed to clarify the important role of m5C-associated gene polymorphisms of neuroblastoma and the underlying mechanisms. We designed a case-control study of children from Jiangsu Province to determine the association between NSUN5 gene polymorphisms and neuroblastoma susceptibility in the Chinese population. However, we did not detect a significant effect on neuroblastoma susceptibility for the NSUN5 rs1880948 A>G polymorphism. Further stratified analysis showed that this SNP had no significant association with the age, sex, tumor primary site, or clinical stage.

This study explores the potential association between the RNA modification gene NSUN5 polymorphism and susceptibility to neuroblastoma, which has not been previously reported. However, there are some limitations to this study. On the one hand, our study population is limited to neuroblastoma patients and volunteers in Jiangsu province, China. It is important to conduct further research on different regions and ethnic groups to explore the NSUN5 polymorphism in various populations. On the other hand, the sample size of SNPs in this study is relatively small. Future research needs to either increase the sample size of the single-center studies or conduct multi-center studies to explore the relationship between this SNP and neuroblastoma.

## Conclusion

In conclusion, we indicate that there is no association between the m5C-modified gene NSUN5 rs1880948 A>G polymorphism and neuroblastoma susceptibility in specific populations. It is worthwhile to pursue further studies that encompass expanded sample sizes and rigorously investigate additional potential functional SNPs. It is valuable to identify other m5C-modified gene SNPs associated with neuroblastoma, possibly providing theoretical implications for potential mechanisms and early diagnosis of neuroblastoma.

## Data Availability

The original contributions presented in the study are provided in the article/supplementary materials; all the data are available upon request from the correspondence authors (Jing He, Yan Zou or Tianyou Yang).
